# The relevance of the LEADS framework during the COVID-19 pandemic

**DOI:** 10.1177/08404704211033002

**Published:** 2021-09-09

**Authors:** Graham Stewart Dickson, Deanne Taylor, Elizabeth Hartney, Bill Tholl, Kelly Grimes, Ming-Ka Chan, John Van Aerde, Tanya Horsley, Ellen Melis

**Affiliations:** 1 Royal Roads University, Canadian Health Leadership Network, Vancouver, British Columbia, Canada.; 2 Interior Health Authority, Kelowna, British Columbia, Canada.; 3 Royal Roads University, Victoria, British Columbia, Canada.; 4 Canadian Health Leadership Network, Ottawa, Ontario, Canada.; 5 University of Manitoba Faculty of Health Sciences, Winnipeg, Manitoba, Canada.; 6 Canadian Society of Physician Leaders, Ottawa, Ontario, Canada.; 7 Royal College of Physicians and Surgeons of Canada, Ottawa, Ontario, Canada.; 8 Unlimited Potentialities, Canadian Health Leadership Network, Mumbai, Maharashtra, India.

## Abstract

COVID-19 has created a unique context for the practice of leadership in healthcare. Given the significant use of the LEADS in a Caring Environment capabilities framework (LEADS) in Canada’s health system, it is important to document the relevancy of LEADS. The authors reviewed literature, conducted research, and reflected on their own experience to identify leadership practices during the pandemic and related them to LEADS. Findings are presented in three sections: Hindsight (before), Insight (during), and Foresight (post). We profile the issue of improving long-term Care to provide an example of how LEADS can be applied in crisis times. Our analysis suggests that while LEADS appears to specify the leadership capabilities needed, it requires adaptation to context. The vision Canada has for healthcare will dictate how LEADS will be used as a guide to leadership practice in the current context or to shape a bolder vision of healthcare’s future.

## Introduction

The COVID-19 pandemic created a unique context for the practice of leadership in the health sector. Prior to COVID-19, the LEADS in a Caring Environment capabilities framework (LEADS) was used extensively in Canada to guide to practice health leadership for maximum impact.^1-3^ But how can LEADS, designed in non-crisis times, guide leadership through the complex challenges the COVID-19 pandemic created—and beyond?

Drawing on a recent study^4^ and literature (note 1), this article will outline three perspectives: (1) *Hindsight*: what LEADS was intended to do and has done; (2) *Insight*: observing and exploring leadership practice during the pandemic and relating them to LEADS; and (3) *Foresight*: suggesting future leadership perspectives and practices that are important for health leaders to consider moving out of the pandemic—and their relation to LEADS. Throughout we apply the pandemic leadership lessons in the context of Long-Term Care (LTC) experience in Canada through the lens of LEADS.

Healthcare has been characterized as a complex adaptive system^2,5^ and requires leaders to use best practices (simple context), good practices (complicated contexts), emergent practices (complex contexts), and more rarely, novel practices (chaotic contexts), as appropriate.^6,7^ Exploring how a framework informs how leadership practices in such rare contexts enriches our broader understanding of health leadership.

### Hindsight

The LEADS framework was developed by practicing leaders^8,9^ and has a strong research base including three validating studies,^10-12^ numerous articles,^13-21^ and two books.^2,22^ It was developed to describe the leadership capabilities needed to respond to major change, characterized by a shift from a provider- to a patient-centred care model, regionalization of services, technological advances, demographic shifts, burgeoning chronic disease, increased use of social media, and ever-growing public and consumer expectations.^10^

LEADS consists of five domains and 20 capabilities (see Table 1). It assists healthcare leaders to effectively lead in personal, interpersonal, operational, and strategic contexts (self to system).^2^ The framework articulates “what” constitutes good leadership for all situations (ie, common vocabulary), but leaves the “how” it is applied to be adapted to context and situation. It has been adopted widely across Canada.^1^

**Table 1.** The LEADS in a Caring Environment capabilities framework

Lead Self (LS): Self-motivated leaders…
*Are self-aware* Aware of their assumptions, values, principles, strengths, and limitations *Manage themselves* They take responsibility for their own performance and health *Develop themselves* They actively seek opportunities and challenges for personal learning, character building, and growth *Demonstrate character* They model qualities such as honesty, integrity, resilience, and confidence
Engage Others (EO): Engaging leaders…
*Foster development of others* They support and challenge others to achieve professional and personal goals *Contribute to the creation of healthy organizations* They create engaging environments where others have meaningful opportunities to contribute and ensure that resources are available to fulfill their expected responsibilities *Communicate effectively* They listen well and encourage open exchange of information and ideas using appropriate communication media *Build teams* They facilitate environments of collaboration and co-operation to achieve results
Achieve Results (AR): Goal-oriented leaders…
*Set direction* They inspire vision by identifying, establishing, and communicating clear and meaningful expectations and outcomes *Strategically align decisions with vision, values, and evidence* They integrate organizational missions, values, and reliable valid evidence to make decisions *Take action to implement decisions* They act in a manner consistent with the organizational values to yield effective, efficient public-centred service *Assess and evaluate* They measure and evaluate outcomes, compare the results against established benchmarks, and correct the course as appropriate
Develop Coalitions (DC): Collaborative leaders…
*Purposefully build partnerships and networks to create results* They create connections, trust, and shared meaning with individuals and groups *Demonstrate a commitment to customers and service* They facilitate collaboration, cooperation, and coalitions among diverse groups and perspectives aimed at learning to improve service *Mobilize knowledge* They employ methods to gather intelligence, encourage open exchange of information, and use quality evidence to influence action across the system *Navigate socio-political environments* They are politically astute. They negotiate through conflict and mobilize support
Systems Transformation (ST): Successful leaders…
*Demonstrate systems/critical thinking* They think analytically and conceptually, questioning and challenging the status quo, to identify issues, solve problems, and design and implement effective processes across systems and stakeholders *Encourage and support innovation* They create a climate of continuous improvement and creativity aimed at systemic change *Orient themselves strategically to the future* They scan the environment for ideas, best practices, and emerging trends that will shape the system *Champion and orchestrate change* They actively contribute to change processes that improve health service delivery

#### Hindsight: LTC

COVID-19 struck those living in LTC facilities first. Over 80% of the deaths due to COVID-19 during the first two waves were among those over the age of 65. The LTC system was already under pressure.^23,24^ We could and should have seen this coming, says André Picard when he refers to the “horror show” of COVID-19: “It took the coronavirus pandemic to open our eyes to the deplorable state of so many of the nation’s long-term care homes: the inhumane conditions, overworked and underpaid staff, and lack of oversight.”^25^ LTC staffing is influenced by system issues and policies,^26^ and reports have identified a system that is underfunded, under tremendous pressure to meet demand, functioning within a lack of transparency and unpredictable funding, and has out-of-date infrastructure.

Leadership factors at the system (design, funding, evaluation), organization (interpretation of standards, staffing, presence of lack of organizational learning systems), and interpersonal (degree of empowerment and autonomy of personal support workers)^27^ levels often made a difference to the care in LTC^28^ and the rates of COVID spread.^29^

## Insight

A crisis is “an unstable or crucial time or state of affairs in which a decisive change is impending.”^30^ The pandemic has become a chronic crisis. Geerts (2020) argues tyat crises have four stages: preparation (before a crisis); the peak; the wake after the peak; and following a crisis.^31^ Yet it is clear that “the peak” of COVID-19 is ongoing and becomes a perpetual peak. In our view, traditional views of leadership in times of crisis need to be adapted to this unique and challenging context, transforming the long-standing norms of healthcare.^20,32,33^

“The virus has no mercy: it exposes all our weaknesses, both individually and societally.”^34^ These include clinician burn out,^35^ glaring deficiencies in pandemic preparedness,^36^ Canada’s extended care standards,^37^ mental health delivery systems,^38^ and inclusion and diversity, including racism.^27,39,40^ It highlights the dangers of misinformation^41^ and supply chain inefficiencies.^42^ Conversely, virtual care^43^ and receptivity to innovation^44^ have accelerated, technology has effectively integrated the workplace with home^45^ and delivered impactful learning.^46^ The imperative of engaging patients, families, and the public^47^ in overcoming “wicked problems”^48^ has been recognized.

In this context, *leadership matters*.^49^ How can LEADS guide the increasingly chaotic and complex leadership contexts that the COVID-19 pandemic has presented?^50^ A recent CHLNet study—*Leading Through COVID*—provides some insight. One senior leader shared: “I was fortunate enough:…I’ve got enough resilience; I wasn’t anxious. I was applying all the stuff that…LEADS has taught me…in the many years that I’ve used it.…and those capabilities gave me the tools and the confidence to just deal with the situation.”^51^ (note 2).

The pandemic has revealed the necessity of caring and compassion for patients, citizens, and staff, reinforcing the “Caring” ethos of the framework. The *Lead Self* domain, focusing on self-awareness and self-management, helps us to deal with heightened emotions in self and others; emphasizes the importance of being aware of biases when promoting equity, diversity, and inclusion; and reinforces the importance of making decisions according to what benefits the community collectively.

The *Engage Others* domain of LEADS emphasizes the need to advocate for personal protective equipment; to address diversity and inclusion issues, and to support self, clinicians, and staff through their own mental challenges: that is, providing the compassion needed to care for COVID-19 patients; coping with unrelenting workloads and stresses on family life; and dealing with feelings of moral distress, fear, loss, and grief. The need for increased visibility and frequent, honest, and supportive communication—deep listening—surfaced.

*Achieve Results* outline the early need for fast decision-making and the rational operational steps to make efficient supply chains, implement vaccination rollouts; and deal with therapeutic and diagnostic backlogs. *Develop Coalitions* relate directly to the challenge of building cohesive and meaningful relationships between federal and provincial governments, between Indigenous communities and health authorities, and between LTC and other components of the health system. The capability of *Mobilize Knowledge* is also highlighted, to ensure public information is timely and accurate; and that misinformation is promptly corrected. The *Encourage and Support innovation* capability of *Systems Transformation* has also been highlighted.

*Demonstrate Systems and Critical Thinking* (*Systems Transformation*) surfaced as an important leadership capability. It includes recognition of when the need for solutions shifts from a chaotic context, with fast decision-making to institute system-wide interventions that bring some order to the chaos (ie, wash your hands, stay home if sick, keep physical distancing) to a complex context, and the ability to surface differing perspectives and a comfort with uncertainty (ie, including a broader circle of stakeholders).^7,52^

### Insight: LTC

The most recent and perhaps profound insights into the LTC leadership challenges come from the April 30, 2021, Final Report of the Ontario Long Term Care COVID-19 Commission.^37^ The report highlights that “strong leadership proved critical in the face of unprecedented challenges,” emphasizes that “leadership does not operate in a vacuum,” and that “quality care depends on effective leadership and accountability.”

Seven characteristics of effective leadership are identified in the report. There is strong alignment with LEADS across all five domains (LS, EO, AR, DC, and ST are acronyms for the LEADS domains; Table 1). Specifically, effective leaders:

1. Create a sense of urgency (ST, Champion and Orchestrate change)
2. Act decisively (AR, Take Action to Implement Decisions)
3. Effectively direct, mobilize, and support staff (AR, Set Direction; EO, Build Teams)
4. Develop and implement creative solutions (AR, Strategically Align Decisions with Vision, Values, and Evidence; ST, Encourage and Support Innovation)
5. Leverage relationships (EO; DC)
6. Demonstrate emotional intelligence and empathy (LS Is Self-Aware, Manages Self; EO Foster Development of Others)
7. Hold self-accountable (LS Demonstrate Character)

The key insights and findings of this Commission clearly reinforce the importance of effective leadership through the COVID crisis and the relevance of LEADS as a useful model for assessing effective leadership and as a model for change needed to address the failings in LTC.

### Foresight

Foresight is “an act of looking forward”—implicit in the LEADS capabilities of *Set Direction* (*AR*) and *Orient Oneself Strategically to the Future* (*ST*). COVID-19 is a catalyst for change. What is its “legacy of leadership” for the Canadian health system?^4,53,54^

We see four primary challenges for healthcare leaders going forward: (1) sustaining affordable, *integrated* health delivery systems; (2) health workforce wellness; (3) envisioning and influencing what the future system will be; and (4) resisting the urge to revert to a version of pre-pandemic normalcy. To address these challenges, future leaders will confront pre-conceived notions of organizational structure, culture, and political dynamics, and their role in causing the deficiencies outlined earlier. Accordingly, effective leaders embrace four overarching principles implicit in the LEADS framework.

The first is to recognize that a heightened *caring ethos* (“Be kind, be calm, be safe”)^55^ is necessary for the leadership actions we take to truly build health workforce wellness and achieve results for the population. Will our leaders be able to provide the required caring and compassion^56^ needed to meet the demands of the ongoing current and post-pandemic phases and create genuinely inclusive systems?

A second overarching principle is to realize the relationship between personal responsibility and accountability for achieving integrated care.^57^ During COVID-19, the standards we count on to guide system performance—accreditation standards,^27^ psychological health in the workplace,^58^ and LEADS itself—did not guarantee the qualities they represent. Leaders must deliberately surface the paradox inherent in the responsibility—accountability relationship: that is, the need to articulate standards to guide practice to meet the needs of the patients and the public on one hand, and the need to be responsible for adhering to those standards when they challenge current practice. We must ask ourselves: Do we believe in our standards, or are they just pro forma statements to satisfy ourselves, politicians, and public?

The third overarching principle is to take a systems^59^ view to redesign and change, reflected most obviously in the domain of *Systems Transformation*. COVID-19 showed us the fragility of artificial boundaries, of sectarianism, of our natural tendencies to see a crisis from a discipline-centric view, as if we could put a wall up between our borders to keep the virus out, and as if parts were independent of the whole. A holistic, learning systems^60^ view challenges that. Leaders need to ask which old, and new, and maybe even not-yet discovered relationships must be built to give voice to perspectives that would otherwise not have been heard during the pandemic and beyond. They need to ground those relationships on trust (*EO* and *DC*). They also need to know how to identify and remove barriers to inefficiency and ineffectiveness (*AR*). Systems thinking also supports the notion that the system we have is the one we are complicit in. The questions for leaders to consider are how they contribute from a systems perspective (*ST*) as well as a personal perspective (*LS*) to the current system and what needs to shift for them to imagine new systems.^2^

A fourth principle is to resist the pressure to “snap back” to the pre-pandemic state. There are many aspects of “normal times” that exert strain on us to revisit past practice rather than persevere with perspectives and practices gained during COVID. Of prime concern is to continue the battle to overcome the health inequities that have surfaced and the importance of continuing to address the systemic patterns of discrimination that were inherent in the pre-COVID world. If we do not resist the subtle drag back to the status quo, leaders will be unable to truly embrace substantive change. Leaders must find ways to *Orient Themselves Strategically to the Future (ST)* if the leadership legacy of COVID-19 is to be realized.

### Foresight: LTC

Better learning from the past (hindsight and insight: as reflected in the “learning systems” guidance from the Systems Transformation domain of LEADS: foresight) can help inform next steps to never again leave Canada’s most vulnerable elderly exposed to the next pandemic. Leadership is required by governments, the public health community, primary and acute health organizations, standard setting bodies (such as Accreditation Canada), and those operating LTC homes. All health leaders must have a deep personal commitment to this change (LS); engage Personal Care Workers in that change in a psychologically safe way (EO); improve operational practices—planning and adherence to effective and up to date LTC standards—within LTC homes (AR). They also need to build the coalitions necessary at the health system level by building stronger relationships with other health partners, unions representing support staff, improved communication with families and loved ones (DC), commensurate with person-centred care models driving healthcare transformation (ST).

### Conclusion

In answer to our question—*how is LEADS relevant in a crisis context*—our analysis suggests that while LEADS appears to specify the leadership capabilities needed, it does so by adopting practices and behaviours to respond to the contextual demands of leadership. Most pre-pandemic LEADS practices were shaped by the existing context. In that context, the capability of *Orients themselves strategically to the future* (ST) suggests small reforms. But a reformed current system is only one future. Should we continue to lead self, engage others, adjust processes to achieve results, and build coalitions, solely within the current context of health service delivery? Our dialogue suggests not. Should we not be envisaging other futures? Transformative futures, Post COVID-19? In those futures, LEADS-based leadership must challenge that existing context. A bolder approach, but still based on relationships, results, and instigating change—self to system as LEADS represents—will be required to create the health system of the future. We invite an ongoing dialogue about what those futures are, and how to operationalize LEADS to create them—together.

## Notes

1. The Canadian Health Leadership Network has been reviewing the literature in healthcare leadership as a regular part of their agenda. They publish select precis of relevant leadership articles in their Top Ten–ten leadership articles drawn from the literature–twice a year; during COVID times, 3 times bi-monthly. Available at https://chlnet.ca/top-ten-reading-lists. Other literature pertinent to the topic of this paper is also included.

2. The CHLNet study used an action research methodology employing grounded theory approaches to data gathering and analysis.
